# Development of an epitope conservancy analysis tool to facilitate the design of epitope-based diagnostics and vaccines

**DOI:** 10.1186/1471-2105-8-361

**Published:** 2007-09-26

**Authors:** Huynh-Hoa Bui, John Sidney, Wei Li, Nicolas Fusseder, Alessandro Sette

**Affiliations:** 1 La Jolla Institute for Allergy and Immunology, Division of Vaccine Discovery, 9420 Athena Circle, La Jolla, CA 92037, USA; 2 Isis Pharmaceuticals, Inc., Antisense Drug Discovery, 1896 Rutherford Road, Carlsbad, CA 92008, USA

## Abstract

**Background:**

In an epitope-based vaccine setting, the use of conserved epitopes would be expected to provide broader protection across multiple strains, or even species, than epitopes derived from highly variable genome regions. Conversely, in a diagnostic and disease monitoring setting, epitopes that are specific to a given pathogen strain, for example, can be used to monitor responses to that particular infectious strain. In both cases, concrete information pertaining to the degree of conservancy of the epitope(s) considered is crucial.

**Results:**

To assist in the selection of epitopes with the desired degree of conservation, we have developed a new tool to determine the variability of epitopes within a given set of protein sequences. The tool was implemented as a component of the Immune Epitope Database and Analysis Resources (IEDB), and is directly accessible at .

**Conclusion:**

An epitope conservancy analysis tool was developed to analyze the variability or conservation of epitopes. The tool is user friendly, and is expected to aid in the design of epitope-based vaccines and diagnostics.

## Background

An epitope can be defined as a group of amino acids derived from a protein antigen that interacts with antibodies or T-cell receptors, thereby activating an immune response. Epitopes can be classified as either continuous or discontinuous. Continuous epitopes, also known as linear or sequential epitopes, are composed of amino acid residues that are contiguous in their primary protein sequence. Conversely, discontinuous epitopes, also known as assembled or conformational epitopes, are composed of amino acid residues that are typically present in different protein regions, but which are brought together by protein folding. Recognition of T cell epitopes typically depends upon processing of antigenic proteins, and as a result T cell epitopes are usually continuous. B cell epitopes, often recognized in the native protein context, may be either continuous or discontinuous.

Pathogenic proteins, in general, and epitopes in particular, are often variable. The degree of variability or similarity of specific proteins or protein regions can provide important information regarding evolutionary, structural, functional, and immunological correlates. Given a set of homologous proteins, phylogenetic relationships can be constructed and used to calculate the evolutionary rate at each amino acid site. Regions that evolve slowly are considered "conserved" while those that evolve rapidly are considered "variable". This approach is widely used in sequence conservation identification and mapping programs such as ConSeq [1] and ConSurf [2,3]. However, to fully describe and characterize protein and/or epitope variability, measures of identity and conservancy are typically utilized. Identity refers to the extent to which two amino acid sequences are invariant, and is measured as the percentage of identical amino acids in the alignment of two sequences. Conservancy is defined as the fraction of protein sequences that contain the epitope considered at or above a specified level of identity. Conversely, the fraction of protein sequences that contain the epitope considered below a specified level of identity reflects the degree of variability or uniqueness of the epitope.

Amino acid residues that are crucial for retention of protein function are believed to be associated with intrinsically lower variability, even under immune pressure. As such, these regions often represent good targets for the development of epitope-based vaccines, as the epitopes targeted can be expected to be present irrespective of disease stage, or particular strain of the pathogen. Furthermore, these same residues are often highly conserved across different related species, such as, for example, has been found in several instances in the context of the poxviridae [4]. As a result, a vaccine containing such conserved epitopes might be effective in providing broad-spectrum protection. Conversely, in a diagnostic and disease monitoring setting, epitopes that are specific to a given pathogen can be used to monitor responses to that particular infectious strain, removing the confounding influence of immune responses derived from previous exposures to partially cross-reactive strains or organisms.

Herein, to assist in the selection of epitopes having a desired level of conservation or, conversely, variability, we have developed an epitope conservancy analysis tool. The tool has been specifically designed to determine the degree of conservation or variability associated with a specific epitope within a given set of protein sequences. Despite our emphasis on epitope identification contexts, it is also apparent that the tool can be utilized for other purposes, such as tracking mutation of epitopes during disease progression. This tool was implemented as a component of the Immune Epitope Database and Analysis Resources (IEDB) [5-7] and was used in predicting the cross-reactivity of influenza A epitopes [8].

## Implementation

### Approach

Given an epitope sequence ***e ***and a set ***P ***of protein sequences {***p***}, our approach is to find the best local alignment(s) of ***e ***on each ***p***. The degree of conservation of ***e ***within ***P ***is calculated as the fraction of {***p***} that matched the aligned ***e ***above a chosen identity level. Two separate processes were developed for assessing the degree of conservation/variability of continuous and discontinuous epitope sequences.

### Continuous sequence

If ***e ***is continuous, the process of finding the best alignment of ***e ***on ***p ***involves breaking ***p ***in to sub-sequences {***s***} of length equal to ***e ***and comparing ***e ***to each ***s***. For a ***p ***sequence of length ***n ***and an ***e ***sequence of length ***m***, a total ***n***-***m***+1 {***s***} different sequences are generated. For each ***e ***and ***s ***comparison, the degree of identity is calculated as a percent of residues that are identical between the two sequences. If ***p ***contains repeat regions, or the identity threshold is low, multiple alignments may be found for ***e***. However, the ***s ***sequence(s) associated with the maximum identity score determines the alignment(s) of ***e ***on ***p***. The degree of conservation of ***e ***is then calculated as the percent of ***p ***sequences in which ***e ***is aligned with an identity level at or above a chosen threshold. Conversely, the degree of variability is calculated as the fraction of ***p ***that ***e ***was aligned below a chosen threshold. An illustrative conservancy analysis of a continuous epitope sequence is shown in Table 1.

**Table 1:** Example conservancy analysis of a continuous sequence

	Reference sequence^1^										Identity^2^	
	
Source	F	L	P	S	D	F	F	P	S	V	No.	(%)
Strain 1	**Y**	L	P	S	D	F	F	P	S	**I**	8	(80)
Strain 2	F	L	P	S	D	F	F	P	S	V	10	(100)
Strain 3	**R**	L	P	S	**K**	**Q**	F	P	S	V	7	(70)
Strain 4	**Y**	**E**	P	**T**	D	F	F	P	S	V	7	(70)
Strain 5	F	L	P	**T**	D	F	F	P	S	V	9	(90)
Strain 6	F	L	P	**T**	D	F	**S**	**F**	**T**	V	6	(60)
Strain 7	F	L	P	S	D	F	F	P	S	V	10	(100)
Strain 8	**Y**	**E**	P	S	**E**	F	**S**	**F**	S	**I**	4	(40)
Strain 9	F	L	P	S	**E**	F	F	P	S	V	9	(90)
Strain 10	F	L	P	S	D	F	F	P	S	V	10	(100)

Total: conservancy at identity threshold ≥ 80%											6	(60)
Total: variability at identity threshold <80%											4	(40)

1. Residues that are different from that of the corresponding residue in the reference sequence are highlighted in bold.

2. Identity indicates the number (%) of residues in the homologous sequence that are identical to the corresponding residue in the reference sequence.

3. Totals indicate the number (%) of strains in which the reference sequence is found with an identity above or below the indicated threshold.

### Discontinuous sequence

If ***e ***is discontinuous, a continuous sequence pattern ***c ***is first generated. For example, given a discontinuous sequence "A1, B3, C6" (meaning A is at position 1, B is at position 3 and C is at position 6), its matching sequence pattern ***c ***is **A**X**B**XX**C **where X is any amino acid residue, and the number of X's between two nearest known amino acid residues is equal to the gap distance between them. Next, the same procedure described for continuous sequences is used to identify the best alignment(s) of ***c ***on ***p***. The identity level is calculated based on the defined epitope residues. An illustration of a discontinuous sequence conservancy analysis is shown in Table 2. To obtain meaningful results, the program only performs calculations for discontinuous sequences consisting of at least three identified residues.

**Table 2:** Example conservancy analysis of a discontinuous sequence

	Reference sequence^1^										Identity^2^	
	
Source	**F**	*X*	*X*	*X*	**D**	**F**	**F**	*X*	*X*	**V**	No.	(%)
Strain 1	**Y**	*L*	*P*	*S*	D	F	F	*P*	*S*	**I**	3	(60)
Strain 2	F	*L*	*P*	*S*	D	F	F	*P*	*S*	V	5	(100)
Strain 3	**R**	*L*	*P*	*S*	**K**	**Q**	F	*P*	*S*	V	2	(40)
Strain 4	**Y**	*E*	*P*	*T*	D	F	F	*P*	*S*	V	4	(80)
Strain 5	F	*L*	*P*	*T*	D	F	F	*P*	*S*	V	5	(100)
Strain 6	F	*L*	*P*	*T*	D	F	**S**	*F*	*T*	V	4	(80)
Strain 7	F	*L*	*P*	*S*	D	F	F	*P*	*S*	V	5	(100)
Strain 8	**Y**	*E*	*P*	*S*	**E**	F	**S**	*F*	*S*	**I**	1	(20)
Strain 9	F	*L*	*P*	*S*	**E**	F	F	*P*	*S*	V	4	(80)
Strain 10	F	*L*	*P*	*S*	D	F	F	*P*	*S*	V	5	(100)

Total: conservancy at identity threshold ≥ 80%											7	(70)
Total: variability at identity threshold <80%											3	(30)

1. Residues that are not defined in the reference sequence are highlighted in italics. Residues that are different from that of the corresponding residue in the reference sequence are highlighted in bold.

2. Identity indicates the number (%) of residues in the homologous sequence that are identical to the corresponding residue in the reference sequence. Residues highlighted with gray shading are not considered in calculating the identity because they are not defined in the reference sequence.

3. Totals indicate the number (%) of strains in which the reference sequence is found with an identity above or below the indicated threshold.

### Program description

**Figure 1:**
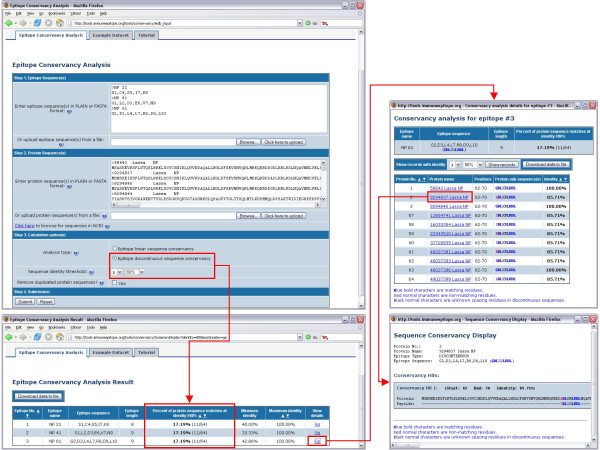
An overview of the epitope conservancy analysis tool.

The epitope conservancy analysis tool was implemented as a Java web-application. An overview of the tool is shown in Figure 1. As input, the program requires the user to provide an epitope set, consisting of one or more epitope sequences, and a set of protein sequences against which each epitope is compared to determine conservancy. Based on our experience, to achieve the best results it is recommended that the protein sequence set utilized be constructed such that redundancies are eliminated and the representation of different substrains and serotypes is balanced. To assist in assembling protein sequence sets, a "Browse for sequences in NCBI" link is provided. When this link is selected, a browser is opened, enabling the user to search for all available protein sequences in NCBI, grouped by organism taxonomic level. To reduce redundancies in the protein sequence set, the user can check the box at the bottom of the input form to have the program automatically remove all duplicated sequences in the protein data set used in the analysis. As output, the program will calculate the fraction of protein sequences that match each epitope sequence above or below a given identity level. The program also calculates the minimum and maximum matching identity level for each epitope. A position mapping of epitope sequences to matching protein sub-fragments is also provided and can be viewed by clicking on the "Go" link in the "View details" column. Detailed sequence mappings of an epitope to all protein sequences in a dataset are also generated. In some cases, if a protein sequence has significant repeat regions, or the level of matching identity is set at a low value, multiple matching protein sub-fragments can be found for a given epitope sequence. All calculation results can be downloaded as text files by clicking on the "Download data to file" button.

## Results and discussion

To determine the degree of conservation of an epitope within a given set of protein sequences, it is necessary to align the epitope to each protein sequence. The degree of conservation is then calculated as the fraction of protein sequences that match the aligned epitope sequence above a defined identity level. Conversely, the degree of variablity is calculated as the fraction of protein sequences that match the aligned epitope sequence below a defined identity level. For continuous epitopes, existing sequence searching and alignment tools, such as BLAST [9] or ClustalW [10], can be used to perform pair-wise local alignment of the epitope to a protein sequence. But, to be relevant in an immunological context, it is crucial that the entire epitope sequence is completely aligned with absolutely no gaps. This requirement entails the use of somewhat different parameters making it cumbersome to use currently existing alignment tools for the characterization of immune epitopes. At the same time, there is no alignment tool currently available for analyzing discontinuous sequences. To rectify these shortcomings, we have developed a robust, user-friendly, epitope conservancy analysis tool. The tool has the capacity to simultaneously align and assess the degree of conservation/variability of each epitope, and can perform these functions for both linear and discontinuous peptide epitope sequences.

For the purpose of developing cross-reactive vaccines that aim toward highly variable pathogens, the use of conserved epitopes across different species is desired. Nevertheless, care should be taken to avoid selecting epitopes that are conserved between the pathogen and the host as this could lead to undesirable induction of auto-immunity. Moreover, extremely conserved epitopes between species are sometimes less immunogenic because they may be derived from proteins that resemble similar proteins in the host. As a result, they are less likely to be recognized by T cells due to self-tolerance. It should also be emphasized that conservation at the sequence level does not assure that the epitope will be equally recognized and cross-reactive. This is due to the differences in the antigen sequences from which the epitope is derived. For T cell epitopes, whether they will be processed in the first place is determined by flanking residues that are different for different antigens. Therefore, the same epitope sequence from different antigens may or may not be generated to subsequently presented and recognized by T cell receptors.

In the case of B cell epitopes, their recognition by an antibody is dependent on the antigen 3D structures. A sequence-wise conserved epitope may not be structurally conserved as it can adopt different conformations in the context of the antigen structures. Exposed amino acids as opposed to buried amino acids are more important in determining the immunogenic of a given segment of peptide. It is because only exposed residues, as observed in antigen:antibody co-crystals, can form contacts with the complementarity determining regions (CDRs) of the corresponding antibody. Those residues that are recognized by a single antibody are often defined as a discontinuous epitope. The epitope conservancy analysis tool developed here can be used to assess the pattern conservation of discontinuous epitopes. Nevertheless, pattern-wise conserved discontinuous epitopes may not be cross-reactive due to the unknown influence of neighboring and inter-dispersed amino acids. As a result, if antigen structures are available, it may be better to predict cross-reactivity based on the epitope's 3D structural conservation.

Depending on the specific needs of a user, an analysis of epitope conservancy may need to be performed at various phylogenetic levels. For example, to determine the potential of a given epitope to be cross-reactive amongst different isolates of a pathogen, or with different microorganisms associated with different pathogenicity, it may be necessary to determine conservancy within a given sub strain, type or clad, within a specific species, or within a genus, or other higher phylogenetic classification group. This type of analysis was utilized previously to identify highly conserved HBV derived epitopes [11,12], and also applied to identify HCV, P. falciparum and HIV derived epitopes [[13], [14], [15], [16], [17], [18], [19]]. Alternatively, to develop epitope-based diagnostic applications aimed at detecting all isolates of a given pathogen but not isolates from related strains, or aimed at detecting specific strains or isolates, it might be necessary to identify epitopes that are highly conserved in only a single or just a few isolates, and poorly conserved in others. Finally, the analysis of potential homologies with sequences expressed by a pathogen's host, or an animal species to be used as an animal model, might be of particular relevance. We anticipate that its relevance might range from predicting poor responses due to self-tolerance and differential performance in animal species expressing different degrees of similarities with a given epitope, to predicting potential safety problems and autoreactivity linked to cross-reactive self reactivity and molecular mimicry. For each of these broad applications, the analysis tool we have developed provides the means to easily assemble the protein sets required to undertake the appropriate analyses, and generates the information necessary to make the appropriate design decisions.

## Conclusion

To address the issue of conservation (or variability) of epitopes or, more broadly speaking, peptide sequences, we have developed a tool to calculate the degree of conservancy (or inversely, the variability) of an epitope within a given protein sequence set. Conservancy can be calculated following user defined identity criteria, and minimal and maximal levels of conservancy are identified. Furthermore, the program provides detail information for each alignment executed. This epitope conservancy analysis tool is publicly available and can be used to assist in the selection of epitopes with the desired pattern of conservation for designing epitope-based diagnostics and vaccines.

## Availability and requirements

• **Project name: **Epitope Conservancy Analysis

• **Project home page: **

• **Operating system(s): **Platform independent

• **Programming language: **Java

• **Other requirements: **Java 1.4 or higher, Tomcat 4.0 or higher

• **License: **none

• **Any restrictions to use by non-academics: **none

## Abbreviations

**BLAST: **Basic Local Alignment Search Tool

**CDRs: **Complementarity determining regions

**IEDB: **Immune Epitope Database and Analysis Resources

**MSA: **Multiple sequence alignment

**NCBI: **National Center for Biotechnology Information

## Competing interests

The author(s) declares that there are no competing interests.

## Authors' contributions

HHB developed the program. WL and NF participated in programming tasks. HHB, JS and AS wrote the manuscript. All authors read and approved the final version.
